# The utility of 68Ga-pentixafor PET/CT in superselective adrenal artery embolization(SAAE) for treating aldosterone adenomas

**DOI:** 10.1007/s00259-025-07465-y

**Published:** 2025-08-15

**Authors:** Fang Zhoufei, Zhang Qixiang, Zhou Wei, Lin Jinxiu, Peng Feng, Cai Han

**Affiliations:** 1 Department of Geriatrics, the First Affiliated Hospital of Fujian Medical University, Fujian Medical University, Fujian Fuzhou, People’s Republic of China; 2 Department of Cardiology, Binhai Campus of the First Affiliated Hospital, National Regional Medical Center, Fujian Medical University, Fuzhou, People’s Republic of China; 3 Fujian Hypertension Research Institute, The First Affiliated Hospital of Fujian Medical University, Fuzhou, People’s Republic of China; 4 Clinical Research Center for Geriatric Hypertension Disease of Fujian province, The First Affiliated Hospital of Fujian Medical University, Fuzhou, People’s Republic of China; 5 Branch of National Clinical Research Center for Aging and Medicine, The First Affiliated Hospital of Fujian Medical University, Fujian, Fuzhou, People’s Republic of China; 6 Department of Geriatrics, National Regional Medical Center, Binhai Campus of the First Affiliated Hospital, Fujian Medical University, Fujian Fuzhou, People’s Republic of China; 7 Department of Nutrition, The First Affiliated Hospital of Fujian Medical University, Fujian Fuzhou, People’s Republic Of China; 8 Department of Cardiology, The First Affiliated Hospital of Fujian Medical University, Fuzhou, Fujian 350005 People’s Republic Of China; 9 Clinical Research Center for Metabolic Heart Disease of Fujian Province, The First Affiliated Hospital of Fujian Medical University, Fujian Fuzhou, People’s Republic of China; 10 The Higher Educational Key Laboratory for Cardiovascular Disease of Fujian Province, The First Affiliated Hospital of Fujian Medical University, Fujian Fuzhou, People’s Republic of China; 11 Key Laboratory of Metabolic Heart Disease in Fujian Province, the First Affiliated Hospital, Fujian Medical University, Fuzhou, China

**Keywords:** 68Ga-Pentixafor, CXC chemokine receptor 4, Superselective adrenal artery embolization, Aldosterone-producing adenoma

## Abstract

**Purpose:**

Expression of CXC chemokine receptor 4 (CXCR4) has proved to be a valuable tool for guiding the diagnosis and treatment of aldosterone-producing adenoma (APA). In this study, we evaluated whether CXCR4 imaging with ^68^Ga-pentixafor PET/CT shows significant changes after superselective adrenal artery embolization (SAAE).

**Methods:**

We prospectively recruited 25 patients with clinically diagnosed APA. All patients were examined with ^68^Ga-pentixafor PET/CT and adrenal venous blood sampling (AVS) before and after SAAE. PET/CT showed that the tracer uptake of unilateral nodular adrenal gland was higher than that of normal adrenal tissue. AVS showed that the dominant secretory side was consistent with that on PET/CT. All patients were successfully treated with SAAE. Clinical follow-up was carried out according to primary aldosteronism surgical outcome (PASO) criteria, and included monitoring of drug type, blood pressure, serum potassium, and aldosterone/renin ratio to evaluate surgical effect. Post operation ^68^Ga-pentixafor PET/CT and the maximum standardized uptake value (SUVmax) were used to observe the uptake of adrenal lesions after SAAE.

**Results:**

Among the 25 APA patients who successfully underwent SAAE, 14 were men and the average age was 51.88 ± 8.89 years. The consistency between AVS and ^68^Ga-pentixafor PET/CT reaches 100%. Before operation, the SUVmax of the diseased side (16.79 ± 2.51, *n* = 25) was significantly higher than that of the non-diseased side (4.56 ± 0.57, *P* < 0.01). According to PASO criteria, 13 of 25 patients achieved complete clinical remission, 9 achieved partial remission and the treatment was ineffective for three patients. 19 cases achieved biochemical complete remission and 3 cases achieved partial remission. Following the treatment, 22 patients showed complete or partial remission. The ^68^Ga-pentixafor SUVmax of the diseased side decreased significantly (16.75 ± 2.54 vs. 4.37 ± 1.52, *n* = 22, *P* < 0.001). For the patients who ineffective to the treatment, the SUVmax did not change (17.07 ± 2.72 vs. 16.17 ± 2.72, *n* = 3, *P* = 0.842). The 25 patients were divided into two groups according to the average value (≥ 65% and < 65%) of the decrease in SUVmax. The decrease in SUVmax correlated with a good patient prognosis under the PASO standard (*P* = 0.009).

**Conclusion:**

A decrease in SUVmax is related to the prognosis. CXCR4 imaging with ^68^Ga-pentixafor can be used for pre- and post-SAAE evaluation in patients with APA.

## Introduction

Hypertension includes primary hypertension and secondary hypertension. Secondary hypertension refers to hypertension with a definite cause, which can be cured or obviously relieved when the cause is controlled. The most common form of secondary hypertension is primary aldosteronism (PA) [1]. PA is a clinical syndrome characterized by hyperaldosteronism, low renin activity, hypertension, and hypokalemia due to excessive aldosterone synthesis in adrenal cortex [2]. Compared with essential hypertension, the risk of cardiovascular and cerebrovascular events, target organ damage, and even the incidence of atrial fibrillation and sleep apnea, are significantly increased in PA patients [3–5]. Early diagnosis and treatment of PA means better blood pressure control, less target organ damage, and better clinical outcomes [6].

The two main types of PA are adrenal aldosterone-producing adenoma (APA, 30%) and idiopathic hyperaldosteronism (IHA, 60%) [2]. The subtype diagnosis, which relies on adrenal computed tomography (CT) and adrenal venous sampling (AVS), determines the treatment strategy. For PA patients with unilateral dominant aldosterone secretion, surgical treatment is the preferred option, whereas medical therapy is more suitable for those with bilateral equal dominance. Although multidisciplinary guidelines worldwide recommend AVS as the reference standard to determine the presence of lateralized aldosterone hypersecretion, its success rate is only approximately 80% [7–9]. Furthermore, a large number of patients, such as those with severe cardiopulmonary dysfunction and obesity, refuse or cannot tolerate laparoscopic surgery. Therefore, the diagnosis, subtype classification, and treatment of PA require further improvements and advancements.

CXCR4 demonstrates characteristic expression in solid tumors, including APA and IHA [10]– [11]. Recent studies used CXCR4 as a probe for ^68^Ga-pentixafor positron emission tomography (PET)/CT imaging in patients with primary aldosteronism who were to undergo laparoscopic surgery. After surgery, hypertension, hyperaldosteronism, and low renin activity may be corrected in some patients [12]. This indicates that ^68^Ga-pentixafor PET/CT can guide the typing diagnosis of patients with primary aldosteronism. However, the consistency between ^68^Ga-pentixafor PET/CT and AVS requires further study.

Adrenal microwave ablation and superselective adrenal artery embolization (SAAE) are recent interventional methods for PA [13–15]. They are both invasive interventions for patients who refuse or cannot tolerate laparoscopic surgery or aldosterone receptor antagonists [16]– [17]. SAAE involves entering the blood-supplying artery of an adrenal aldosterone tumor through an upper limb artery or femoral artery by superselective catheterization, and then injecting absolute alcohol or a coil through a microcatheter to completely block or destroy the blood supply from the artery.

Laparoscopic adrenalectomy (total or partial) enables pathological confirmation, with postoperative follow-up typically conducted via adrenal CT. At present, many experts have raised concerns about the lack of pathological tissue and imaging evaluation methods to reflect adrenal function in SAAE. The current practice of relying solely on peripheral blood electrolytes and aldosterone-to-renin ratio (ARR) lacks sufficient diagnostic reliability. Therefore, we investigated the innovative use of CXCR4 ^68^Ga-Pentixafor PET/CT imaging for evaluating changes in adrenal structure and function before and after SAAE.

## Materials and methods

### Study population

**Fig. 1:**
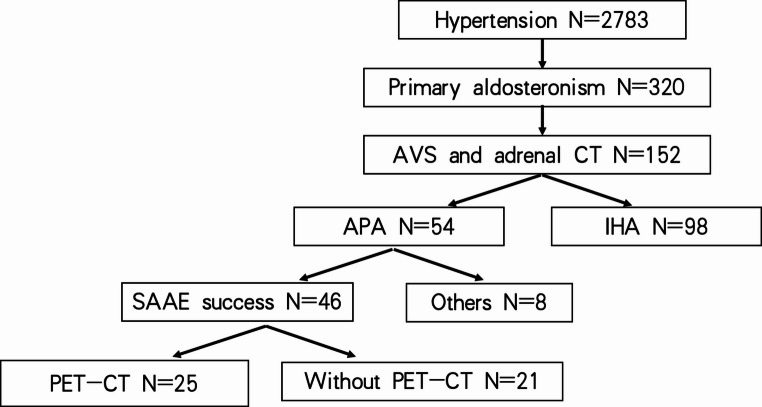
Flowchart of the study design

During 2021–2023, our center diagnosed 320 cases of PA among 2783 confirmed hypertensive patients via saline loading and captopril challenge tests. Among them, 152 patients underwent subtype diagnosis through AVS combined with adrenal CT, which revealed 54 cases of APA and 98 cases of IHA. In the APA group, 46 patients underwent SAAE and the remaining 8 cases (“other” category) included 7 treated with laparoscopic surgery or medications and 1 with a suboptimal SAAE outcome. Notably, 25 SAAE-treated patients completed pre- and post-operative PET/CT examinations (Fig. 1).

The patients were followed up from 5 to 12 months (average 6.92 ± 1.91 months). The medical record collection for the included population was approved by our institution’s ethics committee (approval number [2021] 311). This study is part of a prospective cohort study that is registered in clinical trials.gov with the registration number NCT05188872.

#### Inclusion criteria

(1) Patients diagnosed with PA according to the standards and flowchart developed by the 2020 Working Group on Endocrine Hypertension of the European Society of Hypertension [18]; (2) age between 18 and 70 years, provision of signed informed consent form, and agreement to accept adrenal gland ^68^Ga-Pentixafor PET/CT and AVS; (3) adenoma diameter of less than 2 cm; and (4) patients who chose SAAE following consultation on various treatment schemes including laparoscopic surgery, aldosterone receptor antagonist, and SAAE.

#### Exclusion criteria

(1) patients with acute coronary syndrome, cerebrovascular accident, severe peripheral vascular disease, or major surgical history within one month; (2) terminal stage of malignant tumor; (3) severe blood system diseases; (4) pregnancy, nursing, or planning to get pregnant; (5) severe allergic reaction to iodine-based contrast agent; (6) severe renal insufficiency with serum creatinine > 176 µmol/L; (7) participation in other clinical research or drug research; and (8) unwilling or unable to undergo follow up.

### Clinical indexes

(1) The baseline age, sex, height, weight, body mass index, and course of hypertension were recorded. (2) The baseline serum biochemical indexes, including creatinine, uric acid, total cholesterol, triglyceride, low density lipoprotein cholesterol, and glycosylated hemoglobin were recorded; (3) The left atrial diameter, left ventricular end diastolic diameter, interventricular septum thickness, and left ventricular ejection fraction were recorded on baseline echocardiography. (4) Systolic blood pressure, diastolic blood pressure, antihypertensive drugs, serum potassium, standing serum aldosterone (chemiluminescence method), serum renin concentration (chemiluminescence method), and ratio of aldosterone to renin (ARR) were recorded at baseline and 12 months after SAAE. (5) Common perioperative adverse events of AVS and SAAE, such as fever (body temperature > 38 °C), pain controlled by oral drugs, pleural effusion, pancreatitis, and death, were recorded.

### AVS procedure

**Fig. 2:**
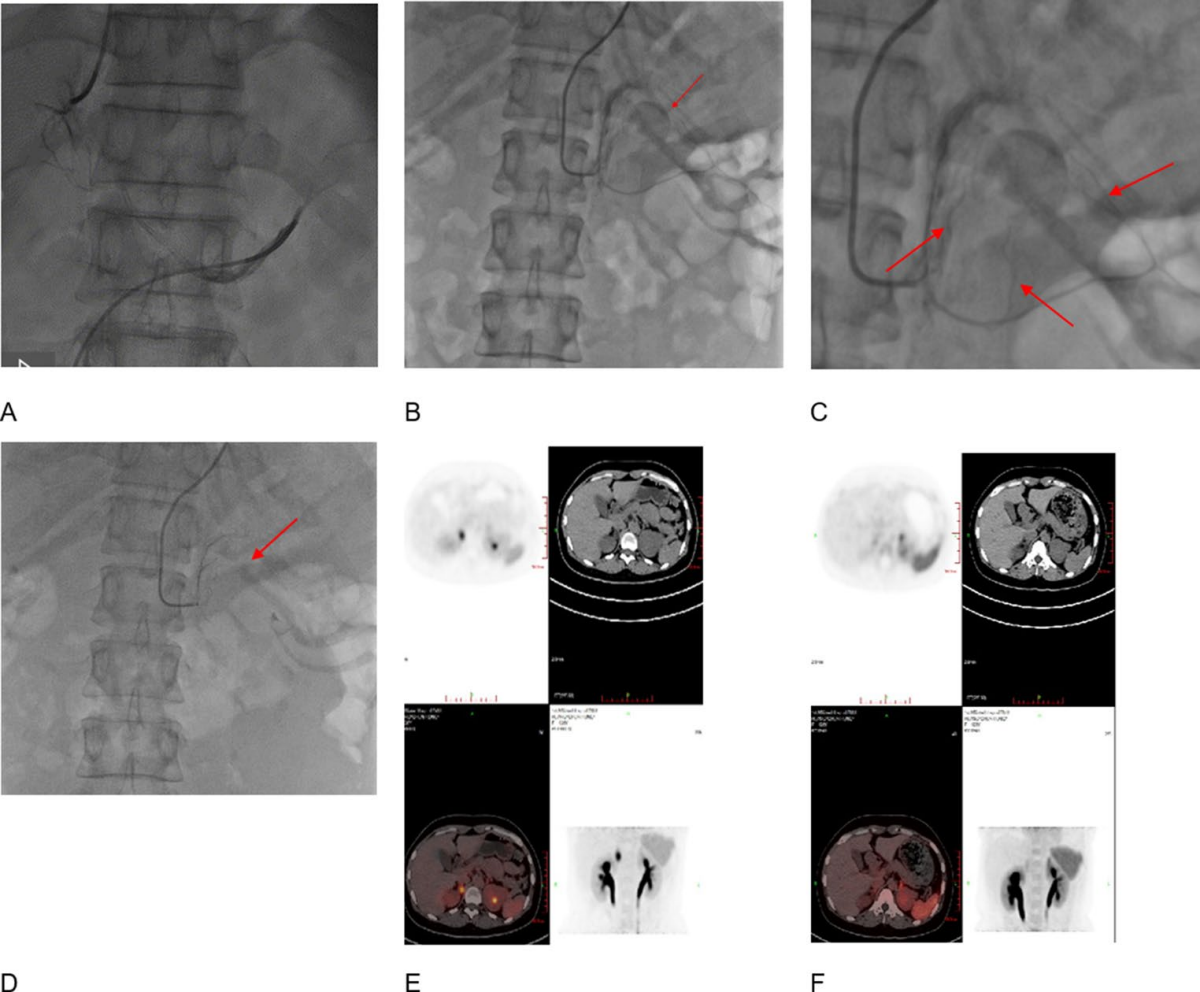
Diagram of adenoma under DSA and CXCR4 imaging with 68Ga-Pentixafor PETCT. **A**: Dual-path AVS procedure; **B**: SAAE procedure. Tig catheterization showed an adrenal adenoma on the left side (arrow). **C**: Local magnification of the image reveals the adenoma supplying artery (arrow); **D**: The tumor structure was not developed after SAAE; **E**: CT and fusion images revealed significantly increased activity at left adrenal lesion with SUVmax = 19.4.; **F**: One year follow-up after SAAE showed a slight radioactivity uptake in the left adrenal with SUVmax = 4.6

Dual-path AVS was performed by two skilled operators working simultaneously. The right cubital vein and right femoral vein were punctured after disinfection and anesthesia, and 5 F sheaths were inserted. An X-ray was performed on the patient’s anterior chest and an F-type MPA catheter (Cordis, USA) was inserted into the inferior vena cava to collect a 2-ml adrenal vein blood sample. Operators simultaneously delivered 5 F MPA catheters (Cordis, USA) into bilateral adrenal veins and then injected a small amount of contrast agent to determine the location. Two 2-ml adrenal venous blood samples were taken simultaneously (Fig. 2a).

The selectivity index (SI) was used to assess the adequacy of the adrenal vein cannulations and was calculated as Cortisol_adrenal vein_/Cortisol_periphrral vein_. Successful AVS was defined as SI > 2 bilaterally. A lateralization index > 2 was defined as dominant secretion.

All procedures were performed without adrenocorticotropic hormone stimulation, were completed before 10 AM local time, and were conducted by two experienced specialists.

### SAAE procedure

SAAE was completed by two doctors with a rich experience in interventional surgery. On postoperative day 1 after AVS, SAAE can be performed as soon as the test results of AVS are obtained.

After disinfection and local anesthesia, the right brachial artery was punctured, an indwelling 6 F arterial sheath (Aipute Company, China) was placed, and 3000 U of heparin were injected into the sheath. The three arteries supplying the adrenal glands included the superior adrenal artery (which is a branch of the inferior phrenic artery), the middle adrenal artery (which is a direct branch of the abdominal aorta), and the inferior adrenal artery (which is a branch of the renal artery). Each of these arteries divides into many branches, which enter the adrenal envelope like a comb and form a network of arteries underneath the envelope. On digital subtraction angiography (DSA) images, adrenal arteriography shows images of the structure and size of the adenoma. The tip of a TIG catheter (Aipute Company, China) was sent into the opening of the affected renal artery under fluoroscopy and renal artery and adrenal artery angiography were performed. A run-through guide wire (Telmao Co., Ltd., Japan) was sent to the distal end of the adrenal artery supplying blood to the adrenal lesions, and a microcatheter (Aipute Company, China) was sent along the guide wire. Contrast agent was injected through the OTW balloon or microcatheter to ensure its superselective placement at the lesion site in the adrenal artery. Anhydrous alcohol was injected slowly through the OTW balloon or microcatheter at an injection speed of 0.5–1.0 mL/minute, and contrast agent was injected into the TIG catheter at the same time, with the total injection amount adjusted according to the distribution of contrast agent in the lesion (generally 1.5–3.0 mL). For adrenal arterial branches exceeding 30 mm in length, coil embolization was performed to ensure permanent vascular occlusion. Adrenal arteriography was then reviewed 5–10 min after the injection of anhydrous alcohol (Fig. 2b-d).

### ^68^Ga-Pentixafor PET/CT scans and image analysis

PET scans were performed on hybrid PET/CT scanners (Biograph mCT 64, Siemens Healthineers, Germany). All imaging was performed at 30–50 min after injection of 106.5 ± 29.4 MBq ^68^Ga-Pentixafor. Non-contrast CT images were acquired over the adrenal region (tube voltage 120 kV, effective tube current 70–120 mA [CareDose 4D, Siemens], 1.0-mm slice thickness, rotation time 0.5 s, and pitch index 0.8). PET images of the upper abdomen and adrenal region (5 min/bed) were acquired immediately after the CT scan. Images were reconstructed using the ordered subset expectation maximization method (2 iterations, 21 subsets, Gaussian filter, 5 mm in full width at half maximum, 200 × 200 image size) and transferred to a Multimodality Workstation (syngo™ MI, Siemens Healthineers). The maximum standardized uptake value (SUVmax) was automatically calculated and used to quantify tracer uptake.

The ^68^Ga-Pentixafor PET/CT images were interpreted by two experienced board-certified nuclear medicine physicians (K.X. Lin and Z.Y. Chen) who were blinded to the clinical information of the patients. The SUVmax of the adrenal lesions were measured by assigning a 10-mm-diameter spherical volume of interest to the area of suspicious uptake. A positive PET/CT lesion detection was defined by visual analysis: adrenal nodule(s) shown on CT (SUVmax ≥ 11) had higher uptake than that of ipsilateral and contralateral normal adrenal gland. Lesions with uptake equal to or less than that of normal adrenal gland on visual assessment were considered negative (Fig. 2e-f).

### Follow-up assessment and outcomes

The clinical and biochemical results were evaluated according to the Primary Aldosteronism Surgical Outcome (PASO) criteria [19]. PASO study consisted of two parts. First, using the Delphi method, we were able to reach consensus for criteria for six outcomes (complete, partial, and absent success of clinical and biochemical outcomes). According to these criteria, complete success is defined as remission, partial success as improvement, and absent success as persistence. Clinical success was defined as normal blood pressure without the help of antihypertensive medication. Partial clinical success was defined as the same blood pressure as before treatment with less antihypertensive drugs or a decrease in blood pressure after treatment with the same amount or less antihypertensive medication. Absence of clinical success was defined as unchanged or elevated blood pressure with either the same amount or an increase in antihypertensive medication. Complete biochemical success was defined as correction of hypokalemia and normalization of ARR. Partial biochemical success was defined as correction of hypokalemia and increased ARR (compared with pre-treatment), with ≥ 50% decrease in baseline plasma aldosterone concentration. Absence of biochemical success was defined as persistent hypokalemia or persistent raised ARR (compared with pre-treatment) with < 50% decrease in baseline plasma aldosterone concentration.

### Statistical analysis

SPSS 24.0 was used for statistical analysis. Continuous variables are expressed as mean ± SD (age, height, weight, systolic blood pressure, diastolic blood pressure, cholesterol, triglycerides, low-density lipoprotein cholesterol, serum creatinine, uric acid, and glycated hemoglobin). Aldosterone, renin, and ARR are expressed as median with interquartile range. Categorical data are shown as number (n) and percentage (%). For continuous data, the paired-samples *t*-test was used to make comparisons between two groups. Non-parametric tests were performed on measurement data with a non-normal distribution. The chi-square test and Fisher exact test were used to compare categorical data. All tests were two-tailed with 95% confidence intervals (CI), and P values < 0.05 were considered statistically significant.

## Results

### Baseline and follow-up characteristics

Of the 25 patients diagnosed with APA, 14 were men and the average age was 51.88 ± 8.89 years. Preoperative ^68^Ga-Pentixfor PET/CT showed that the SUVmax of the diseased side (16.79 ± 2.51, *n* = 25) was significantly higher than that of the non-diseased side (4.56 ± 0.57, *P* < 0.001). (Table 1)

**Table 1:** Baseline and follow-up characteristics

	Baseline(*N* = 25)	follow-up(*N* = 25)	*P* value
Male(%)	14(56.0)	/	/
Age(years)	51.88 ± 8.89	/	/
Weight(Kg)	69.56 ± 13.14	/	/
Course of hypertension(years)	7.08 ± 6.76	/	/
SBP(mmHg)	161.60 ± 18.12	123.20 ± 9.53	< 0.001
DBP(mmHg)	96.16 ± 10.24	72.12 ± 1.24	< 0.001
Antihypertensive drugs	2(2,2.5)	0(0,1)	< 0.001
Cholesterol (mmol/L)	4.14 ± 1.01	4.16 ± 0.88	0.79
Triglyceride (mmol/L)	1.28 ± 1.12	1.43 ± 0.49	0.33
LDL (mmol/L)	2.82 ± 0.95	2.72 ± 0.81	0.13
Creatinine (µmol/L)	73.68 ± 18.80	78.70 ± 18.37	0.07
Uric acid(mmol/L)	311.04 ± 123.97	349.08 ± 90.79	0.08
Glycosylated hemoglobin (%)	5.76 ± 1.19	5.96 ± 0.67	0.28
LAD(mm)	3.96 ± 0.46	3.97 ± 0.40	0.73
LVEDD(mm)	5.00 ± 0.52	4.96 ± 0.46	0.21
IVST(mm)	1.09 ± 0.20	1.08 ± 0.15	0.72
LVEF(%)	68.00 ± 4.81	65.98 ± 4.66	0.06
Serum potassium(mmol/L)	3.13 ± 0.54	4.01 ± 0.60	< 0.001
Supine ALD (ng/dl)	24.40(14.44,38.85)	/	/
Supine DRC (µIU/ml)	1.10(0.50,1.70)	/	/
Supine ARR (ng/dl)	82.55(22.30,134.29)	/	/
Upright ALD (ng/dl)	23.10(15.66,43.35)	14.50(8.50,18.05)	< 0.001
Uprigh DRC (µIU/ml)	2.60(1.00,4.55)	6.20(3.80,11.65)	< 0.001
Upright ARR (ng/dl)	29.50(7.72, 57.32)	1.79(1.07,3.09)	< 0.001
Adenoma size(mm)	1.21 ± 0.27	/	/
Non-diseased side SUVmax	4.56 ± 0.57	4.41 ± 0.53	0.19
Diseased side SUVmax	16.79 ± 2.51	5.79 ± 4.24	< 0.001

*SBP *systolic blood pressure, *DBP* diastolic blood pressure, *LDL* low-density lipoprotein cholesterol, *LAD* left atrial diameter, *LVEDD* left ventricular end diastolic dimension, *IVST* interventricular septum thickness, *LVEF* left ventricular ejection fraction, *ALD* serum aldosterone, *DRC* serum renin concentration, *ARR* aldosterone-to-renin ratio. Age was calculated in years from birthdate to date of informed consent

### Analysis of AVS parameters and adverse events

AVS was successfully performed in all patients, with SUVmax ≥ 11 as the tangent point. Using SUVmax ≥ 11 as the cutoff point, all patients underwent successful AVS. The consistency between AVS and ^68^Ga-pentixafor PET/CT reaches 100%. There was no significant difference in SI, operation duration, or adverse events between left-side and right-side adenoma. (Table 2)

**Table 2:** Parameters and adverse events of AVS

	Left Adenoma (*N* = 11)	Right Adenoma (*N* = 14)	*P* value
SI (left)	45.32 ± 61.26	33.27 ± 30.35	0.53
SI (right)	43.71 ± 49.03	43.53 ± 39.76	0.99
Contrast agent(ml)	30.00 ± 5.00	28.93 ± 7.98	0.70
Times(min)	6.64 ± 1.69	7.07 ± 2.46	0.62
Complication			
Fever	0	1	1.00
Adrenal artery hematoma/dissection	0	0	1.00

*SI* Selectivity index

### Analysis of SAAE parameters and adverse events

SAAE was successfully performed in 25 patients. In one patient, the adrenal artery was too small for microcatheters to enter, resulting in embolization failure. The right adrenal artery mostly originates from the renal artery, while the left adrenal artery mostly originates from the abdominal aorta. Adenomas were located on the left or right side, and there were no significant differences in renal adenoma supplying arteries, alcohol use, operation duration, or adverse events between the two sides. (Table 3)

**Table 3:** Parameters and adverse events of SAAE

		Left Adenoma (*N* = 11)	Right Adenoma (*N* = 14)	*P* value
Adrenal artery opening	Aorta abdominalis opening(N)	7	4	0.09
	Renal artery opening(N)	5	12	0.04
the number of blood vessels supplying the adenoma(N)		2.00(2,2)	2.00(2,2.25)	0.81
Total anhydrous alcohol(ml)		2.59 ± 0.49	2.35 ± 0.60	0.31
Contrast agent(ml)		69.55 ± 14.40	74.29 ± 18.59	0.49
Times(min)		26.64 ± 2.50	26.14 ± 3.28	0.68
Complication				
Fever		3	4	1.00
Pain		9	12	1.00
Pleural effusion		1	1	1.00
Pancreatitis		/	/	/
Death		/	/	/
SUVmax		14.99 ± 2.79	16.63 ± 2.36	0.73

### Prognostic analysis

Clinical and biochemical outcomes were evaluated according to PASO criteria. Of the 25 patients who successfully received SAAE, 13 achieved clinical complete remission, 9 achieved partial remission, and SAAE was ineffective in 3. Among patients who responded effectively to SAAE treatment, 19 achieved biochemical complete remission, while 3 attained partial remission. According to the PASO criteria, unilateral adrenalectomy for primary aldosteronism can result in six possible outcomes, with 3 cases classified as treatment failure (no remission). The results showed that in the 22 patients who achieved complete or partial remission, the SUVmax of 68Ga-Pentixfor PET/CT on the affected side significantly decreased after SAAE (16.75 ± 2.54 vs. 4.37 ± 1.52, *n* = 22, *P* < 0.001). In contrast, the three non-responding patients exhibited no significant change in SUVmax (17.07 ± 2.72 vs. 16.17 ± 2.72, *n* = 3, *P* = 0.842) (Table 4).

**Table 4:** Clinical and biochemical outcomes

	complete success and partial success	absent success	*P* value
SUVmax before	16.75 ± 2.54	17.07 ± 2.72	0.84
SUVmax after	4.37 ± 1.52	16.17 ± 2.72	< 0.001
△SUVmax	12.38 ± 3.00	0.90 ± 1.49	< 0.001
SUVmax descend range(%)	73.23 ± 9.67	5.13 ± 9.30	< 0.001

### Correlation between ^68^Ga-pentixafor PET/CT and PASO criteria

The 25 patients were divided into two groups according to the average decrease (65%) in SUVmax: decrease in SUVmax of ≥ 65% and < 65%. Decrease in SUVmax correlated with a good patient prognosis under the PASO standard (*P* = 0.009). (Table 5)

**Table 5:** Clinical and biochemical outcomes

	Complete success + partial success	Absent success
SUVmax descend range ≥ 65%(N)	19	/
SUVmax descend range<65%(N)	3	3

## Discussion

This paper mainly focuses on the study of PA, using CXCR4-targeted imaging with ^68^Ga-pentixafor PET/CT to investigate the changes before and after SAAE. For the first time, we integrated this novel CXCR4-based imaging approach into the diagnosis and treatment paradigm for primary aldosteronism. CXCR4, with its unique biological properties and distribution patterns, is an ideal molecular target for non-invasive visualization of disease processes. By utilizing the advanced technical method of CXCR4 imaging with ^68^Ga-Pentixafor PET/CT and comparing it with the reference standard AVS recommended by guidelines, we conducted an in-depth exploration of the utility of ^68^Ga-Pentixafor PET/CT in patients who underwent SAAE for the treatment of aldosterone adenomas.

The results were compelling: a remarkable reduction in SUVmax was observed in adrenal tissue on the affected side of patients who achieved complete or partial remission postoperatively. When CXCR4-targeted imaging was used to evaluate patients’ prognoses, it provided a quantitative advantage over traditional imaging techniques. Decrease in SUVmax negatively correlated with patient outcomes, which vividly demonstrates the quantitative superiority of CXCR4-targeted imaging in prognostic assessment, and further highlights the crucial role of CXCR4-targeted imaging. Our findings not only confirm that ^68^Ga-Pentixafor PET/CT with CXCR4 as its targeted biomarker can be effectively used to evaluate patients with aldosterone-producing adenomas (APA) before and after SAAE, but also strongly validate the efficacy and safety of SAAE in APA patients. Thus, this study underscores the indispensable value of CXCR4-based imaging for advancing the management of primary aldosteronism.

SAAE is a specialized surgical intervention, and evaluation of its prognostic value is important. Assessing the prognosis following SAAE serves as a cornerstone for accurately gauging the treatment’s efficacy and forecasting the long-term health status of patients. Currently, the existing assessment approach relies predominantly on the PASO criteria and encompasses both biochemical and clinical remission, but it does have a major limitation: the lack of an imaging-related component. This is where the chemokine receptor CXCR4 becomes an important factor. As a key molecular target endowed with unique biological characteristics, CXCR4 has remarkable potential for non-invasive targeted imaging. In stark contrast to traditional imaging modalities, CXCR4-based imaging using ^68^Ga-Pentixafor PET/CT provides the distinct advantage of visualizing disease processes at the molecular level, thereby providing more accurate information on pathologic changes.

Historically, the outcomes of laparoscopic adrenalectomy have been evaluated through CT imaging. Some research demonstrated that prognostic assessments integrating AVS and the PASO criteria were more robust for patients with primary aldosteronism (PA). However, unfortunately, a large number of patients do not undergo the reference standard AVS prior to surgery. This omission has led to a wide disparity in prognostic evaluations across different cases, undermining the consistency and accuracy of such assessments.

Our center has more than ten years of experience in interventional diagnosis and treatment of hypertension, which helps to ensure the success rate of AVS and SAAE. For this study, we chose patients with APA for the following two reasons. First, prior studies by our team, along with those of other leading experts [17, 20], have firmly established the efficacy of SAAE for managing both APA and IHA. However, compared with IHA patients, APA patients treated with SAAE showed significantly better clinical results. This difference may be attributed to the unique anatomical characteristics of APA. On DSA, APA typically presents as a well-defined round or oval tumor structure with particularly prominent feeding arteries. This clear visualization of the tumor and its blood supply facilitates precise embolization, ultimately leading to more favorable treatment results. Second, under physiological conditions, the average SUV of the adrenal gland ranges from approximately 2–4, generally not exceeding 5. In stark contrast, APA is characterized by an exceptionally high SUVmax, whereas IHA shows only a marginal increase in SUVmax in comparison with normal adrenal tissue. Consequently, ^68^Ga-pentixafor PET/CT imaging targeting CXCR4 has relatively low specificity and sensitivity when used to evaluate IHA. Given the current limitations of imaging technology, expanding the research cohort to include all PA patients would likely introduce significant biases into the study results. Since this study represents the first application of ^68^Ga-pentixafor PET/CT-based CXCR4 imaging to assess the efficacy of SAAE, focusing on APA patients is more conducive to observing positive results.

The proportion of patients achieving clinical complete remission (13 out of 25) was notably lower than the number attaining biochemical complete remission (19 out of 25). Among the 22 patients who achieved complete or partial remission, 21 experienced a reduction in SUVmax exceeding 50%, with an average decrease of 65%. According to the PASO criteria, SUVmax is strongly correlated with positive patient outcomes, which not only establishes SUVmax as a reliable prognostic indicator, but also demonstrates the importance of CXCR4 imaging as a predictor of response to therapy. CXCR4 imaging provides a quantitative measure that can be used to monitor disease progression, assess treatment efficacy, and guide clinical decision making in real-time. In this study, three patients showed no response to the interventional surgery, which may be because of chronic hypertension. Hypertension due to renal impairment and atherosclerosis may not be reversible even if secondary causative factors are excluded. It is essential to acknowledge that SAAE still harbors several limitations. First, the precise correlation between the embolized blood vessels and the secretory function of the adenoma remains elusive. Second, the adrenal arteries are exceptionally slender, measuring only 1–2 mm in diameter, and their orifices lack a consistent location. This anatomical peculiarity substantially heightens the risk of incomplete embolization during the procedure. Third, determining whether the adenoma receives blood supply from non-adrenal arteries during surgery poses a considerable challenge. Fourth, there exists the possibility of recanalization of the embolized blood vessels, which may undermine the long-term efficacy of the treatment. On a positive note, SAAE offers a plethora of notable advantages. Its minimally invasive characteristic, cost-effectiveness, reduced hospital stays, and minimal blood loss render it an attractive treatment option. Presently, SAAE stands as an efficacious therapeutic modality for patients who are either averse to, or ineligible for, traditional surgery, as well as those who experience adverse effects from aldosterone receptor antagonists.

CXCR4 is a G protein-coupled receptor that is widely distributed in cell membranes [10]. There is a significant correlation between CXCR4 and aldosterone synthase expression [11]. As a specific ligand of CXCR4, ^68^Ga-pentixafor provides functional PET/CT imaging through its specific binding to CXCR4 receptors on the cell membrane, which has been explored in the diagnosis and treatment of PA [21] (1). CXCR4 imaging with ^68^Ga-pentixfor PET/CT can be used for the early screening of patients who are highly suspected to have PA, and its sensitivity and specificity are higher than those of CT [22]. (2) AVS is the reference standard, but is an invasive examination that requires hospitalization. The success rate varies considerably from operator to operator, making it difficult to perform. Amir Hossein Chaman Baz and others confirmed that CXCR4 imaging with ^68^Ga-pentixfor PET/CT was not inferior to AVS in respect to the choice of PA treatment [23]. (3) Ding et al. showed that SUVmax was positively correlated with lesion diameter and negatively correlated with preoperative blood potassium level. SUVmax was significantly higher in patients who were cured after laparoscopic surgery than in those who improved after surgery [12].

## Summary

Although this study is limited by its single-center design and small sample size, it successfully reveals the value of CXCR4-targeted imaging in the diagnosis and treatment of PA, while outlining future research directions and clinical application prospects.

1. Future Research Directions
  Optimization of precision diagnosis: investigate the optimal SUVmax threshold for CXCR4 imaging using ^68^Ga-pentixafor PET/CT. This aims to break through the limitations of traditional imaging (which only shows anatomical structures) and leverage differences in CXCR4 molecular expression to achieve early and precise diagnosis of APA.
  Upgrading of treatment monitoring: conduct in-depth research on the long-term follow-up value of this imaging technique for PA patients receiving drug therapy, providing molecular-level evidence of efficacy for the development of personalized treatment plans.
2. Existing research achievements
  Validation of diagnostic reliability: the high consistency between ^68^Ga-pentixafor PET/CT and the reference standard of AVS in the evaluation of APA fully confirms the clinical reliability of CXCR4-targeted imaging.
  Innovation in treatment evaluation: significant changes in SUVmax before and after SAAE in APA patients indicate that this technique can not only visualize tumors, but also dynamically capture functional changes in adenomas at the molecular level. This fills the technical gap in evaluation of SAAE efficacy and provides a more precise and efficient solution for PA diagnosis and treatment.
3. Clinical application prospects
  Although further research is needed to clarify its application in IHA, this study has established CXCR4 as a core biomarker in the diagnosis and treatment of PA. With its advantages of non-invasiveness and molecular visualization, we expect ^68^Ga-pentixafor PET/CT to integrate diagnosis, monitoring, and efficacy evaluation, revolutionizing PA management models and driving PA diagnosis and treatment in a more precise patient-centered direction.

## Data Availability

The data that support the findings of this study are available from the corresponding author upon reasonable request.

## Declarations

The authors have no conflicts to disclose.
